# Neurological complications associated with influenza in hospitalized children

**DOI:** 10.1111/irv.13075

**Published:** 2022-12-13

**Authors:** Watsamon Jantarabenjakul, Tanitnun Paprad, Tunchanok Paprad, Suvaporn Anugulruengkitt, Chitsanu Pancharoen, Thanyawee Puthanakit, Krisnachai Chomtho

**Affiliations:** ^1^ Division of Infectious Diseases, Department of Pediatrics, Faculty of Medicine Chulalongkorn University Bangkok Thailand; ^2^ Center of Excellence for Pediatric Infectious Diseases and Vaccines, Faculty of Medicine Chulalongkorn University Bangkok Thailand; ^3^ Thai Red Cross Emerging Infectious Diseases Clinical Center King Chulalongkorn Memorial Hospital Bangkok Thailand; ^4^ Division of Neurological Diseases, Department of Pediatrics, Faculty of Medicine Chulalongkorn University Bangkok Thailand; ^5^ Division of Diagnostic Radiology, Department of Radiology, Faculty of Medicine Chulalongkorn University, King Chulalongkorn Memorial Hospital Bangkok Thailand

**Keywords:** children, encephalopathy, influenza, neurological complication, seizure

## Abstract

**Background:**

Influenza is a known respiratory and potential neurotropic virus. This study aimed to determine the prevalence and outcomes of influenza‐related neurological complications among hospitalized children.

**Methods:**

All medical records of hospitalized children aged <18 years old diagnosed with influenza at a tertiary care hospital in Bangkok were retrospectively reviewed. Influenza infection was confirmed by rapid antigen or reverse transcription polymerase chain reaction tests. Neurological characteristics and clinical outcomes were analyzed using the Pediatric Cerebral Performance Category Scale.

**Results:**

From 2013 to 2018, 397 hospitalized children with a median age of 3.7 years (interquartile range [IQR]: 1.6–6.9) were included. The prevalence of neurological complications, including seizure or acute encephalopathy, was 16.9% (95% confidence interval [CI]: 13.3–20.9). Influenza A and B were identified in 73.1% and 26.9% of the patients, respectively. Among 39 (58.2%) acute symptomatic seizure cases, 25 (37.3%) children had simple febrile seizures, 7 (10.4%) had repetitive seizures, and 7 (10.4%) had provoked seizures with pre‐existing epilepsy. For 28 (41.8%) encephalopathy cases, the clinical courses were benign in 20 (29.9%) cases and severe in 8 (11.9%) cases. Ten (14.9%) children needed intensive care monitoring, and 62 (93.5%) fully recovered to their baselines at hospital discharge. Predisposing factors to the neurological complications included a history of febrile seizure (adjusted odds ratio [aOR]: 20.3; 95% CI: 6.6–63.0), pre‐existing epilepsy (aOR: 3.6; 95% CI: 1.3–10.2), and a history of other neurological disorders (aOR: 3.5; 95% CI: 1.2–10.2).

**Conclusions:**

One fifth of hospitalized children with influenza had neurological complications with a favorable outcome. Children with pre‐existing neurological conditions were at higher risk for developing neurological complications.

## 1 BACKGROUND

Influenza is a common contagious respiratory virus that primarily causes upper and lower respiratory tract infections. The virus is estimated to infect 9 to 45 million people yearly, mainly young children.^1^ Typical clinical presentations in children include high fever and gastrointestinal conditions such as reduced appetite, nausea, and vomiting.^2^, ^3^ Moreover, influenza can accompany neurological complications in up to 10–30% of pediatric patients.^4^, ^5^, ^6^ The common neurological symptoms in children are seizures and encephalopathy, with variable severities.^7^ In general, seizures are more common^8^; however, in Asian countries, encephalopathy has been reported with increasing frequencies.^9^, ^10^, ^11^ Furthermore, encephalopathy is likely to result in more severe sequelae than febrile seizures.^12^ Other neurological complications include meningitis, stroke, acute disseminated encephalomyelitis (ADEM), and Guillain–Barré syndrome.^7^, ^13^ Various studies reported that the mortality rate varied from 7% to around 30%.^9^, ^14^, ^15^ Though most children experienced no sequelae,^5^, ^7^, ^10^ up to 10% of hospitalized children with influenza might have neurological abnormalities at discharge or up to 40% in cases of severe complications.^5^, ^14^ Clinical sequelae or persistent disabilities can be acute necrotizing encephalopathy (ANE), myelitis, and meningitis.^14^, ^16^, ^17^ In previous studies, possible risk factors included a history of febrile seizure, genetic predisposition in other family members, and pre‐existing neurological disease.^5^, ^13^, ^17^

In tropical countries, seasonal influenza outbreaks can occur annually or biannually, whereas seasonal outbreaks during winter are more common in Arctic countries.^18^ In Thailand, biannual outbreaks were recorded during the rainy and winter seasons, with the major incidence from August to September.^18^ According to Department of Public Health Thailand data, children under 5 years old were at the highest risk of getting influenza, followed by those between 5 and 14 years old. However, there were limited reports on influenza‐associated neurological complications in Thailand, so this study primarily aimed to determine the prevalence and outcomes of neurological complications associated with influenza among hospitalized children and secondarily aimed to assess factors related to influenza‐related neurological complications.

## 2 METHODS

This study was a retrospective medical record review in a tertiary care center, King Chulalongkorn Memorial Hospital (KCMH), Bangkok, Thailand, which has a capacity of 300 beds for children. The study included hospitalized children aged 1 month to less than 18 years old who were diagnosed with influenza from January 2013 to December 2018 and had any neurological signs or symptoms. The cases were identified by the ICD‐10 coding of J09–J11 influenza group, and G02, G04, and G05. All electronic medical records of these patients were reviewed for demographic data, clinical diagnosis, treatment, and outcomes using the Pediatric Cerebral Performance Category (PCPC) Scale.^19^ In addition, cases with neurological complications were retrospectively reviewed by two pediatric neurologists (TaP and KC). The study was approved by the Institutional Review Board (IRB) of the Faculty of Medicine, Chulalongkorn University, and was conducted under the tenets of the Declaration of Helsinki.

Routinely, an influenza test was performed among hospitalized children with respiratory symptoms and/or clinical suspicion for isolation and diagnosis. Influenza infection was diagnosed by rapid antigen or reverse transcription polymerase chain reaction (RT‐PCR) tests within 1 month following the onset of neurological symptoms. The RT‐PCR was performed when the clinical manifestations were likely to be influenza, but the rapid antigen test was negative. The rapid influenza antigen test was the immunochromatography test for influenza A and B antigens (QuickNavi Flu®, Denka Seiken). The RT‐PCR tests were either real‐time RT‐PCR (Simplexa™ Flu A/B & RSV Direct, DiaSorin Molecular LLC, California) or multiplex RT‐PCR with bead hybridization (NxTAG®, Respiratory Pathogen Panel, Luminex, Canada), which were commercially available.

### 2.1 A review of medical records

Data extraction from medical records was as follows: demographic data, admission unit, underlying medical conditions, clinical characteristics of neurological symptoms and other symptoms, a history of influenza vaccination, influenza virus subtypes, treatments, duration of admission, and clinical outcomes at discharge and 6 months after diagnosis, which were assessed by the PCPC Scale^19^, ^20^ (Appendix A). In addition, brain imaging, including computed tomography (CT) and/or magnetic resonance imaging (MRI), was reviewed by a neuroradiologist (TuP).

### 2.2 Case definition of neurological complications

The neurological complications were defined as having either seizure or acute encephalopathy.^7^, ^21^ The seizure was further categorized into a provoked seizure in children with underlying epilepsy and new onset of seizure with the following characteristics: (1) generalized seizure (defined as non‐focal seizures without underlying seizure disorder, brain abnormality, or other significant metabolic diseases), (2) repetitive seizure (defined as multiple seizures), or (3) status epilepticus (defined as continuous seizure activity lasting longer than 5 min for generalized seizure and longer than 10 min for focal seizures).^22^ Acute encephalopathy is characterized by altered mental status or personality change lasting more than 24 h. Encephalopathy severity is classified as benign encephalopathy if the conditions improve with no sequelae and severe encephalopathy if the conditions result in severe impairments of consciousness with residual morbidities or a PCPC Scale of >3. ANE is described using a clinico‐radiological combination of symmetrical lesions in bilateral thalami and the involvement of systemic organs. Additionally, mild encephalitis/encephalopathy with reversible splenial lesion (MERS) is characterized by a transient mild impairment of consciousness and a reversible lesion in the splenium of the corpus callosum on an MRI study.^23^, ^24^ Any children who had a seizure with subsequent alternation of consciousness of more than 24 h were categorized into an acute encephalopathy group.

### 2.3 Statistical analysis

Statistical analysis was performed using stata Version 13.1 (StataCorp, College Station, TX, USA). Continuous variables were described as the median and interquartile range (IQR), and categorical variables were percentages. A chi‐squared test was applied for categorical variables, and a Wilcoxon rank‐sum test was used for continuous variables. Logistic regression was used to determine independent neurological complications. The multivariate model included significant covariates from univariate analysis with p values ≤0.1 and backward stepwise selection. The p value of <0.05 was considered statistically significant.

## 3 RESULTS

### 3.1 Prevalence of neurological complications and the clinical characteristics

From January 2013 to December 2018, 397 hospitalized children with a median age of 3.7 years (IQR: 1.6–6.9) were admitted to our hospital due to influenza (Table 1). The prevalence of influenza‐associated neurological complications was 16.9% (67/397 children, 95% confidence interval [CI]: 13.3–20.9). Most children (193/397, 48.6%) were 1–5 years old, and 44/67 (65.7%) children with neurological complications were in this age group. In the influenza‐associated neurological complication group, 16 children (23.8%) had a history of febrile seizure compared to only 5 (1.5%) who had influenza with no neurological complications. Of 67 cases in the with neurological complication group, 10 (14.9%) children required intensive care unit admission, which was higher than the cases with no neurological complications (4.5%) (p value 0.004). Influenza type (A/B) was similar in both groups with and without neurological complications.

**TABLE 1:** Baseline characteristics of hospitalized children with influenza

Characteristic	All (N = 397)	With neurological complications (N = 67)	No neurological complications (N = 330)	p value
Median age (IQR) (years)	3.7 (1.6–6.9)	2.9 (1.8–5.4)	4 (1.5–7.5)	0.09
Age group, n (%)				0.01
<6 months	21 (5.3)	2 (3.0)	19 (5.8)	
6 months to 1 year	33 (8.3)	3 (4.5)	30 (9.1)	
1–5 years	193 (48.6)	44 (65.7)	149 (45.1)	
>5 years	150 (37.8)	18 (26.8)	132 (40.0)	
Male, n (%)	233 (58.7)	37 (55.2)	196 (59.4)	0.53
History, n (%)				<0.001
Febrile seizure	21 (5.3)	16 (23.8)	5 (1.5)	
Epilepsy	21 (5.3)	8 (11.9)	13 (3.9)	
Other neurological diseases	20 (5.0)	7 (10.4)	13 (3.9)	
Other underlying diseases	204 (51.4)	18 (26.9)	186 (56.3)	
History of influenza vaccination, n (%)	56 (14.1)	14 (20.9)	42 (12.7)	0.08
Influenza type, n (%)				0.42
Influenza A	274 (69.0)	49 (73.1)	225 (68.2)	
Influenza B	123 (31.0)	18 (26.9)	105 (31.8)	
ICU admission, n (%)	25 (6.2)	10 (14.9)	15 (4.5)	0.004
LOS due to influenza, median (IQR)^a^	3 (2–6)	3 (2–5)	3 (2–7)	0.29
Death, n (%)	6 (1.5)	1 (1.5)	5 (1.5)	0.73

Abbreviations: ICU, intensive care unit; IQR, interquartile range; LOS, length of stay.

^a^ Only the patients admitted to the hospital due to influenza. Any children who were diagnosed with influenza after 3 days of admission were excluded from the study because the influenza was considered as nosocomial infection (N = 296).

### 3.2 Clinical characteristics of neurological complications

Table 2 summarizes the neurological complications. The median (IQR) interval between the onset of influenza symptoms to neurological symptoms was 1 (1–2) day. Most children (88%, 59/67) had a high fever (defined as a body temperature ≥38°C). Of the 67 children with neurological complications, 45/67 (67.2%) had seizures, 3 cases had status epilepticus, and 3 cases were defined as encephalopathy. Among 39/67 (58.2%) acute symptomatic seizure cases, 25/67 (37.3%) had simple febrile seizures, 7/67 (10.4%) had repetitive seizures, and 7/67 (10.4%) had provoked seizures in pre‐existing epilepsy. In the encephalopathy group, 20/67 (29.9%) children had acute benign encephalopathy, and 8/67 (11.9%) children had severe encephalopathy. Oseltamivir was the main treatment in 60/67 children (89.6%). At hospital discharge, 62 patients (62/67, 92.5%) had complete recovery at discharge, whereas 3/67 (4.5%) had clinical sequelae and recovered within 6 months. One patient was newly diagnosed with craniopharyngioma and had residual neurological deficits associated with tumor removal. One 5‐year‐old child with a history of encephalopathy suspected of inborn metabolism errors died. She presented with fever, coryza, and alteration of consciousness. Her CT scan showed diffuse brain swelling with leptomeningeal enhancement at bilateral high frontoparietal lobes and maximum ammonia 3264 μg/dl. She was not improved by continuous venovenous hemodialysis for ammonia removal and died within 2 days. Her final diagnosis was ornithine transcarboxylase deficiency.

**TABLE 2:** Clinical characteristics for hospitalized children with influenza with neurological complications (N = 67)

Characteristics	N (%)
Symptoms	
Fever ≥38°C	59 (88.0)
Alteration of conscious	28 (41.8)
Content	5 (7.5)
Level	23 (34.3)
Seizure	45 (67.2)
Status epilepticus	3 (4.5)
Abnormal movement	2 (2.9)
Neck stiffness	2 (2.9)
Focal neurodeficits	1 (1.5)
Clinical syndrome	
Acute symptomatic seizure	39 (58.2)
Single‐episode seizure	25 (37.3)
Repetitive seizure	7 (10.4)
Fever‐provoked seizure in known‐case epilepsy patients	7 (10.4)
Encephalitis/encephalopathy	28 (41.8)
Benign encephalopathy	20 (29.9)
Severe encephalopathy	8 (11.9)
Treatments	
Oseltamivir	60 (89.6)
Corticosteroid	5 (7.5)
Intravenous immunoglobulin (IVIG)	1 (1.5)
Outcome	
Complete recovery to their baseline at discharge	62 (92.5)
Neurological deficits with complete recovery within 6 months	3 (4.5)
Long‐term disability	1 (1.5)
Death	1 (1.5)

Fourteen children (14/67; 20.9%) had a cerebrospinal fluid (CSF) analysis; however, only two cases were evaluated for influenza virus by the RT‐PCR method, and the results were not detected. CSF profiles were either normal or aseptic meningitis profiles in which the median (IQR) of white blood cell count was 3 (2–10)/high‐power field with monocyte predominate, protein level was 20 (14–52) mg/dl, and glucose level was 73 (65–87) mg/dl. Ten children (10/67; 14.9%) underwent brain imaging studies, either CT or MRI (Table 3). The common findings were generalized brain edema (four children) and focal lesions (three children). In addition, electroencephalogram (EEG) was performed in 14 children (14/67; 20.9%). Among eight children with severe encephalopathy, two children had normal EEG findings, and the other two children revealed encephalopathic patterns: the generalized slow‐wave, extreme delta brush, and rhythmic delta activity EEG in one child and continuous low‐amplitude slow wave in another child (Table 3). The EEG findings in the other 10 children with seizures were as follows: Six had normal EEG findings, and the other four had focal epileptiform discharges.

**TABLE 3:** Clinical manifestations and outcomes of six children with severe encephalopathy from influenza

Case and clinical presentation	Influenza type	CSF profiles	Brain CT/MRI findings	EEG findings	Treatments	Outcomes
1. A 7‐year‐old girl with vitiligo ‐ Current medications: Azathioprine, dexamethasone, and laser therapy ‐ Clinical presentation: Fever, drowsiness, and spastic tone (PCPC Grade 5)	A	‐ OP: Cannot be evaluated ‐ CP: 30 mmH_2_O ‐ WBC: 3/HPF ‐ RBC: 405/HPF ‐ Glucose: 89 mg/dl ‐ Protein: 11 mg/dl	‐ MRI: Unremarkable study	‐ Generalized slow wave, no epileptiform discharges repeat extreme delta brush pattern with rhythmic delta activity	‐ Endotracheal intubation for 4 days ‐ Methylprednisolone ‐ IVIG 5 days ‐ Levetiracetam ‐ Phenytoin	‐ PCPC Grade 4 at discharge ‐ Complete recovery to PCPC Grade 1 at 6 months
2. A 2‐year‐old boy with a history of febrile convulsion at 1 year old ‐ Clinical presentation: Fever, generalized tonic–clonic seizure with status epilepticus, and abnormal movement as myoclonus (PCPC Grade 5)	B	‐ OP: N/A ‐ CP: N/A ‐ WBC: 1/HPF ‐ RBC: 0/HPF ‐ Glucose: 85 mg/dl ‐ Protein: 43 mg/dl	‐ CT: Hypoxic ischemic encephalopathy ‐ MRI: Hyperintense T2WI and hypointense T1WI lesion at bilateral thalami and splenium of corpus callosum (Figure 1)	‐ No spike wave	‐ Endotracheal intubation for 14 days ‐ Methylprednisolone ‐ Oseltamivir ‐ Cefotaxime ‐ Acyclovir ‐ Phenytoin ‐ Phenobarbital ‐ Levetiracetam	‐ Final diagnosis: Acute necrotizing encephalopathy ‐ PCPC Grade 4 at discharge ‐ Clinical improvement to PCPC Grade 2 at 6 months with delayed development ‐ MRI result improved at 7 months
3. An 11‐year‐old boy with no underlying medical conditions ‐ Clinical presentation: Fever with cough, rhinorrhea, and alteration of conscious both content and level (PCPC Grade 5)	A	‐ OP: 32 mmH_2_O ‐ CP: 23 mmH_2_O ‐ WBC: 7/HPF ‐ RBC: 0/HPF ‐ Glucose: 101 mg/dl ‐ Protein: 23.4 mg/dl	‐ CT: Generalized brain edema ‐ MRI: Hyperintense foci along cortical surface in bilateral frontoparietal lobes, bilateral cingulate gyri, and bilateral insular cortices, a non‐enhancing ill‐defined oval‐shaped hyperintense lesion with marked restricted diffusion at splenium of corpus collosum (Figure 2)	‐ Not performed	‐ Endotracheal intubation for 3 days ‐ Methylprednisolone for 3 days ‐ Cefotaxime ‐ 3% NaCl	‐ Final diagnosis: MERS ‐ PCPC Grade 1 at discharge
4. A 4‐year‐old boy with chronic idiopathic thrombocytopenic purpura ‐ Clinical presentation: Fever with cough and rhinorrhea, and drowsiness as comatose (PCPC Grade 5)	A	‐ OP: 36 mmH_2_O ‐ CP: 32 mmH_2_O ‐ WBC: 0/HPF ‐ RBC: 0/HPF ‐ Glucose: 74.1 mg/dl ‐ Protein: 13.7 mg/dl	‐ CT: Generalized brain edema	‐ Not performed	‐ Oseltamivir ‐ Cefotaxime ‐ Vancomycin ‐ 3% NaCl	‐ Complete improvement after 24 h ‐ PCPC Grade 1 at discharge
5. A 13‐year‐old girl with no underlying disease ‐ Clinical presentation: Fever with cough and rhinorrhea, ataxia, and horizontal nystagmus to the left (PCPC Grade 4)	B	‐ Not performed	‐ MRI: Well‐defined border lesion was shown at left pontomedullary junction, which is hypointense on T1WI and hyperintense on T2WI/FLAIR. This lesion causes mass effect to the fourth ventricle (Figure 3)	‐ Not performed	‐ Methylprednisolone ‐ Oseltamivir	‐ Final diagnosis: Brainstem leukoencephalopathy ‐ All clinical improvement except limit EOM at discharge ‐ PCPC Grade 2 at discharge and at 6 months ‐ MRI resolved at 3 months
6. A 3‐year‐old with congenital hypotonia ‐ Clinical presentation: Fever with cough and rhinorrhea, alternation of consciousness, and seizure suspected status epilepticus (PCPC Grade 5)	B	‐ Not performed	‐ Not performed	‐ No epileptiform discharge	‐ Endotracheal intubation for 1 day ‐ Oseltamivir ‐ Cefotaxime ‐ Azithromycin ‐ Phenytoin	‐ Clinical improvement after 24 h ‐ PCPC Grade 1 at discharge

Abbreviations: CP, closing pressure; CSF, cerebrospinal fluid; CT, computed tomography; EEG, electroencephalogram; EOM, extraocular movement; FLAIR, fluid‐attenuated inversion recovery; HPF, high‐power field; IVIG, intravenous immunoglobulin; MERS, mild encephalitis/encephalopathy with reversible splenial lesion; MRI, magnetic resonance imaging; N/A, no data availability; OP, opening pressure; PCPC, Pediatric Cerebral Performance Category; RBC, red blood cell; WBC, white blood cell.

**FIGURE 1:**
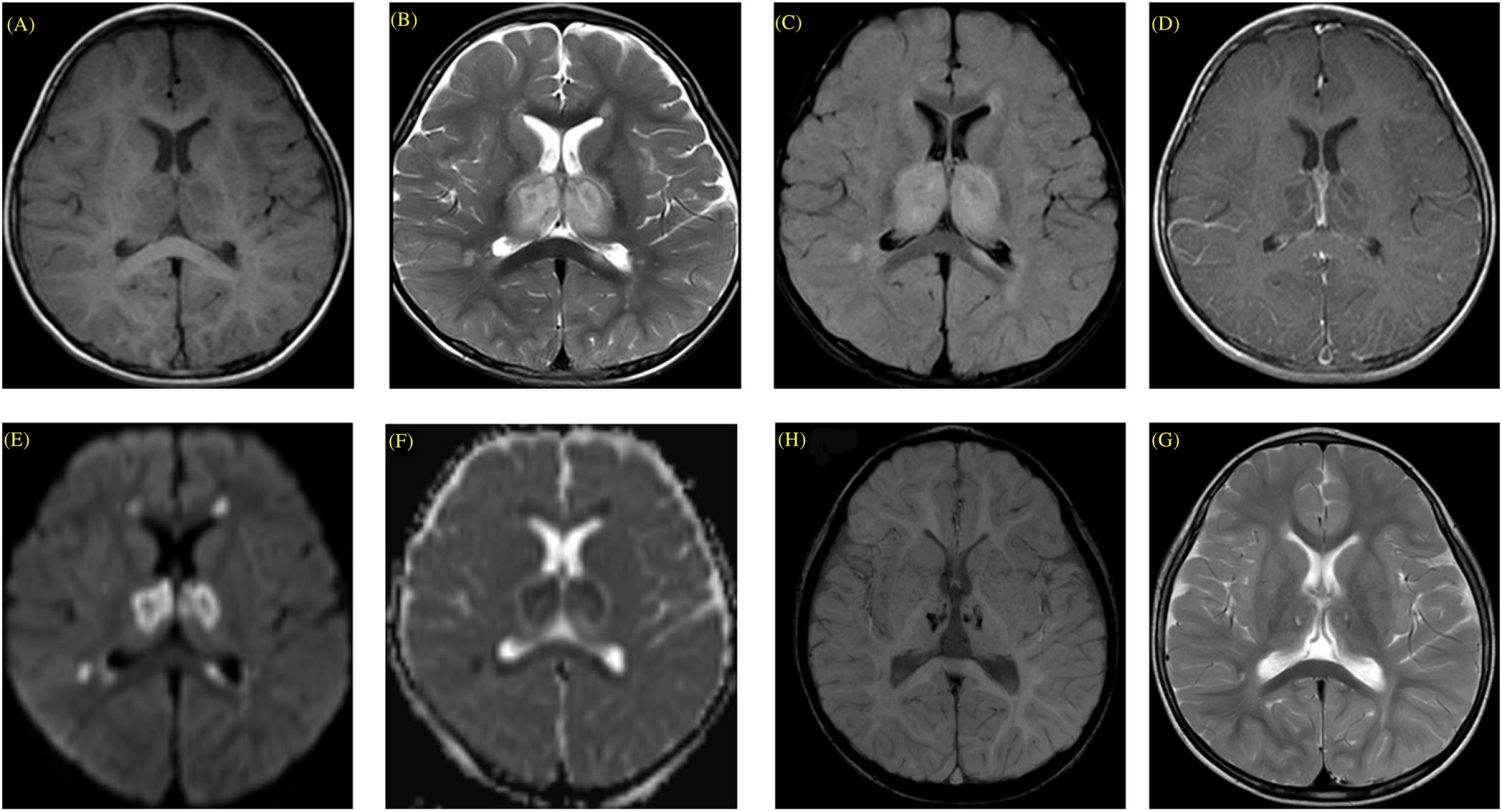
Brain magnetic resonance imaging (MRI) of acute necrotizing encaphalopathy (Case No. 2). (A–F) Initial MRI shows typical brain imaging of acute necrotizing encephalopathy. (A) Axial longitudinal relaxation time (T1)‐weighted imaging (T1WI), (B) transverse relaxation time (T2)‐weighted imaging (T2WI), (C) T2WI/fluid‐attenuated inversion recovery (FLAIR), (D) contrast‐enhanced T1WI, (E) diffusion‐weighted imaging (DWI), and (F) apparent diffusion coefficient (ADC) demonstrate bilateral symmetrical rim‐enhancing thalamic lesions, showing internal T1 hypointensity and T2 hyperintensity with restricted diffusion and central hypointense hemorrhagic foci. Perilesional edema with mild mass effect is seen. These findings correspond with classic target‐like appearance. (G, H) Repeated brain MRI at 7 months after diagnosis, (G) axial T2WI, and (H) susceptibility‐weighted imaging (SWI) show evolutional change of bilateral thalamic lesions to encephalomalacia–gliosis with hemosiderin deposition.

**FIGURE 2:**
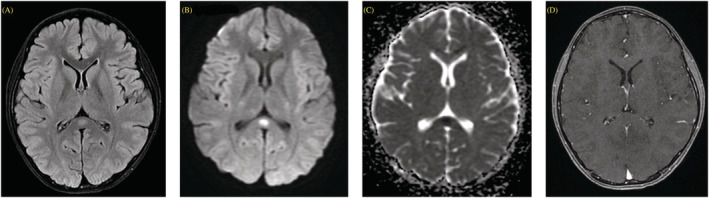
Brain magnetic resonance imaging (MRI) of mild encephalitis/encephalopathy with reversible splenial lesion (MERS) (Case No. 3). (A) Axial longitudinal relaxation time‐weighted imaging (T1WI), (B) transverse relaxation time‐weighted imaging (T2WI)/fluid‐attenuated inversion recovery (FLAIR), (C) diffusion‐weighted imaging (DWI), and (D) apparent diffusion coefficient (ADC) show circumscribed oval‐shaped non‐enhancing T2/FLAIR hyperintense lesion with diffusion restriction involving splenium of the corpus callosum. This finding is typical for cytotoxic lesion of the corpus callosum (CLOCC).

**FIGURE 3:**
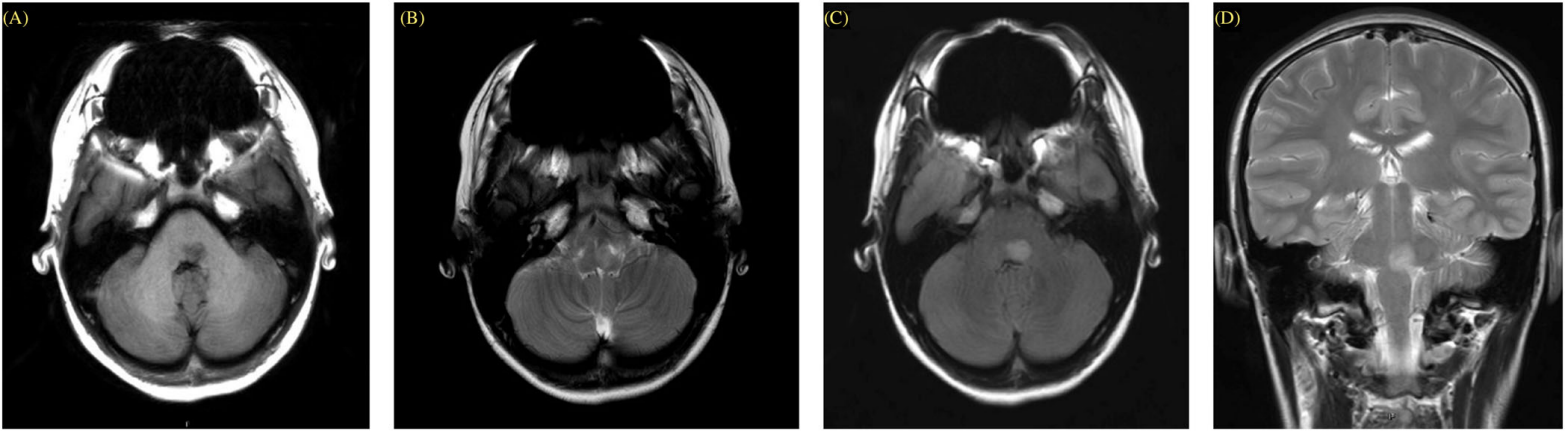
Initial magnetic resonance imaging (MRI) of brain stem leukoencephalopathy (Case No. 5). (A) Axial longitudinal relaxation time‐weighted imaging (T1W1), (B) axial transverse relaxation time‐weighted imaging (T2WI), (C) axial T2/fluid‐attenuated inversion recovery (FLAIR), and (D) coronal T2WI. Well‐defined border lesion was shown at left pontomedullary junction, which is hypointense on T1WI and hyperintense on T2WI/FLAIR. This lesion causes mass effect to the fourth ventricle.

### 3.3 Risk factors for neurological complications

On multivariable analysis, predisposing factors associated with neurological complications included febrile seizure (adjusted odds ratio [aOR]: 20.3; 95% CI: 6.6–63.0), pre‐existing epilepsy (aOR: 3.6; 95% CI: 1.3–10.2), and other neurological disorders (aOR: 3.5; 95% CI: 1.2–10.2). Age, gender, virus type, and vaccination status were not statistically significant (Table 4).

**TABLE 4:** Risk factors associated with neurological complications among hospitalized children with influenza

	Univariate		Multivariate	
	OR (95% CI)	p value	aOR (95% CI)	p value
Age				
<1 year	Ref		Ref	
1–5 years	2.9 (1.1–7.7)	0.03	1.6 (0.6–4.6)	0.36
>5 years	1.3 (0.5–3.8)	0.59	0.9 (0.3–2.6)	0.79
Female vs. male	1.2 (0.7–2.0)	0.53		
Febrile seizure	20.4 (7.2–58.1)	<0.001	20.3 (6.6–63.0)	<0.001
Pre‐existing epilepsy	3.3 (1.3–8.3)	0.01	3.6 (1.3–10.2)	0.02
Neurological diseases	2.8 (1.1–7.4)	0.03	3.5 (1.2–10.2)	0.02
Influenza type				
Influenza A	1.3 (0.7–2.3)	0.43		
Influenza B	Ref			
History of flu vaccination				
No	Ref		Ref	
Yes	2.0 (1.0–4.0)	0.04	1.4 (0.7–3.1)	0.37

Abbreviations: aOR, adjusted odds ratio; CI, confidence interval; OR, odds ratio.

## 4 DISCUSSION

In this study, the prevalence of influenza‐associated neurological complications was 17% of hospitalized children, and the most common features were a simple febrile seizure and benign encephalopathy. However, some children required intensive care units and experienced permanent sequelae. In this study, the typical form of encephalopathy included ANE, MERS, and brainstem leukoencephalopathy. In addition, patients with a history of febrile seizures and other neurological conditions were at greater risk for developing neurological complications.

Previous reports reported that the prevalence of influenza‐associated neurological complications varied between 9% and 48%, comparable with 17% in our study.^4^, ^5^, ^6^, ^25^, ^26^, ^27^, ^28^, ^29^ Our study included only hospitalized children, indicating more severe influenza cases. Therefore, the prevalence of any associated complications was likely higher than in overall influenza children. Moreover, Asian ethnicity is another reported risk factor for developing neurological complications associated with influenza, related to genetic predisposition.^30^, ^31^ However, a cohort study of Caucasian patients reported a relatively similar prevalence (18%) to ours.^26^ The influenza vaccination rate was not significantly different between the children with and without neurological complications. Notably, some children had no fever at presentation, which might delay the diagnosis of influenza infections. Moreover, some influenza children might present with solely neurological symptoms. Therefore, clinical suspicion and awareness of influenza infections were essential, despite the complete typical manifestations.

The clinical manifestations of influenza‐associated neurological complications can vary, from mild symptoms such as headache, numbness, and drowsiness to more moderate and severe symptoms such as seizure and acute encephalopathy.^7^ Moreover, the severity of a seizure can be a single episode of seizure, repetitive seizures, or status epilepticus. The definite pathophysiology of the neurological complications remains unclear, whether they are due to direct viral invasion or immunological or inflammatory responses.^32^, ^33^ In this study, there was no evidence of direct viral invasion from the RT‐PCR for influenza from the CSF, so the children with severe encephalopathy cases were treated with methylprednisolone aiming to mediate the immunological responses.

Typical imaging findings of influenza‐associated neurological complications have been reported using the terms of clinico‐radiological diagnosis.^10^, ^30^, ^31^, ^34^, ^35^, ^36^ In this study, several clinico‐radiological diagnostic terms were applied, including ANE, MERS, and a rare form of isolated brainstem leukoencephalopathy (Table 3). However, there are also some other reported lesions, for example, the lesion in the pons.^37^ Notably, though the aforementioned clinico‐radiological findings have been reported in association with influenza, similar findings can also be found in other infections.^34^, ^38^, ^39^, ^40^ In this study, four patients had electroencephalographic findings of focal epileptiform activities, representing the central nervous system inflammation.

Most children in this study (93%) had complete or nearly complete recovery at discharge. Some children took 6 months following diagnosis to full recovery. One case was diagnosed with ANE and experienced long‐term neurological deficits.^24^, ^34^, ^41^, ^42^ As cytokine storms and genetic mutation in RAN binding protein 2 (RANBP2) gene may be responsible for the pathogenesis of severe encephalopathy, including ANE, the use of methylprednisolone is likely beneficial for treating cytokine storm and metabolic dysfunction by decreasing inflammation, providing protective regulatory effects on mitochondria and limiting the extent of brain edema.^34^, ^43^ In this study, children with acute encephalopathy were treated with methylprednisolone and had good responses. However, mild encephalopathy is generally self‐limited. In this study, two patients had the distinct form of white matter lesions, MERS, and acute brain stem leukoencephalopathy. Patients with MERS were reported to have a good prognosis^36^; however, one child with MERS in our study presented a marked alteration of consciousness with complete recovery. Another rare presentation in our children included acute brain stem leukoencephalopathy, in which her presenting neurological deficits were debilitating. With intravenous methylprednisolone treatment, the child had got complete recovery.

Predisposing factors of influenza‐associated neurological complications included previous neurological disorders such as febrile convulsion, which is the most powerful, epilepsy, and other neurological diseases, consistent with previous studies.^8^, ^10^, ^26^, ^28^ Age was also another important factor. Children less than 5 years old tend to have seizure symptoms more than older ones. Regarding the virus types, influenza A is more associated with neurological complications than influenza B.^44^, ^45^ In our study, 73% of children with neurological complications tested positive for influenza A. Although, after adjusting the odds ratios, the virus type is not a significant risk factor, half of the severe encephalopathy cases in this study tested positive for influenza A. The finding could represent the neurotropic behavior of each type.

Our study reported the clinical variabilities of influenza‐associated neurological complications from common findings such as seizures to rare but unique complications such as ANE, MERS, and acute brainstem leukoencephalopathy. However, there were some limitations in our study. Firstly, the study was a retrospective study that unavoidably contained some selection and information bias. Secondly, the study population was collected from the tertiary care hospital, involving more severe cases. Thirdly, CSF analysis was not performed in every case. Notably, there were no peripheral nervous system complications such as Guillain–Barré syndrome and myositis in our study.

## 5 CONCLUSION

Neurological complications occurred in one fifth of hospitalized children due to influenza. The most common complication was a generalized seizure. Though most had good recovery at 6 months of follow‐up, few cases with encephalopathy experienced morbidity. Due to the significant response to systemic immunosuppressive treatments in some severe neurological complications, early recognition of the clinical syndromes improves treatment outcomes. Children with pre‐existing neurological conditions were at greater risk of developing neurological complications.

## CONFLICTS OF INTEREST

The authors declare no conflicts of interest.

## ETHICS APPROVAL STATEMENT

The study was approved by the Institutional Review Board (IRB) of the Faculty of Medicine, Chulalongkorn University, and was conducted under the tenets of the Declaration of Helsinki.

## AUTHOR CONTRIBUTIONS

**Watsamon Jantarabenjakul:** Conceptualization; data curation; formal analysis; writing‐original draft. **Tanitnun Paprad:** Conceptualization; data curation; formal analysis; investigation; writing‐original draft. **Tunchanok Paprad:** Conceptualization; data curation; formal analysis; investigation; writing‐review and editing. **Suvaporn Anugulruengkitt:** Data curation; writing‐review and editing. **Chitsanu Pancharoen:** Conceptualization; supervision; writing‐review and editing. **Thanyawee Puthanakit:** Conceptualization; methodology; writing‐review and editing. **Krisnachai Chomtho:** Conceptualization; supervision; writing‐review and editing.

### PEER REVIEW

The peer review history for this article is available at https://publons.com/publon/10.1111/irv.13075.

## Data Availability

The data that support the findings of this study are available from the corresponding author upon reasonable request.

## APPENDIX A

**Table:** **TABLE A1** Pediatric Cerebral Performance Category Scale^17^

Score	Category	Description
1	Normal	At age‐appropriate level; school‐age child attends regular school
2	Mild disability	Conscious, alert, able to interact at age‐appropriate level; regular school, but grades perhaps not age appropriate, possibility of mild neurological deficit
3	Moderate disability	Conscious, age‐appropriate independent activities of daily life; special education classroom and/or learning deficit present
4	Severe disability	Conscious, dependent in others for daily support because of impaired brain function
5	Coma or vegetative state	Any degree of coma, unaware, even if awake in appearance, without interaction with the environment; no evidence of cortex function; possibility for some reflexive response, spontaneous eye‐opening, sleep–wake cycles
6	Brain death/death	Brain death, death
